# Immune senescence and exhaustion in breast cancer patients with a history of childhood maltreatment

**DOI:** 10.1016/j.bbih.2026.101345

**Published:** 2026-08-31

**Authors:** Ivon Johanna Rodríguez-Rodríguez, Sara Lucía Neira-Ortíz, Manuela Llano-León, Nicolas Lalinde-Ruiz, Henry Mauricio Parada-Gereda, Ricardo Sanchez-Pedraza, Carlos Alberto Parra-López

**Affiliations:** a Grupo de Inmunología y medicina traslacional, Departamento de Microbiología, Facultad de Medicina, Universidad Nacional de Colombia, Bogotá, Colombia; b Departamento de Movimiento Corporal Humano, Facultad de Medicina, Universidad Nacional de Colombia, Bogotá, Colombia; c Grupo de Investigaciones Clínicas en Cancer, Facultad de Medicina, Universidad Nacional de Colombia, Bogotá, Colombia; d Clínicas colsanitas, Clínica Reina Sofia, Grupo de investigación en nutrición clínica y rehabilitación-keralty, Bogotá, Colombia

**Keywords:** Childhood maltreatment, Immunosenescence, Breast cancer

## Abstract

**Purpose:**

Child maltreatment has lasting adverse effects on both physical and mental health and is associated with reduced quality of life. In Colombia, this problem remains highly prevalent. Childhood maltreatment has been identified as a risk factor for multiple chronic conditions, including cardiovascular disease, autoimmune disorders, cancer, and stress-related psychiatric disorders such as major depressive disorder. Early-life exposure to chronic stress is associated with immunological alterations, including increased production of pro-inflammatory cytokines, and the development of a chronic low-grade inflammatory state. Against this background, we investigated the association between a history of childhood maltreatment, systemic low-grade inflammation, and the distribution of leukocyte populations exhibiting features of senescence in patients with breast cancer.

**Methods:**

We included 20 women diagnosed with breast cancer, who completed the Childhood Trauma Questionnaire-Short Form (CTQ-SF). Peripheral blood was collected to isolate plasma and PBMCs. Plasma cytokines were measured using a Cytometric Bead Array. Markers of immune senescence and exhaustion in monocytes, NK cells, and T cells were analyzed by flow cytometry.

**Results:**

The CTQ-SF assessment revealed a high prevalence of severe-to-extreme childhood maltreatment, with 40% of participants reporting severe physical abuse, 35% severe emotional abuse, and 40% severe physical neglect. Immunologically, CM + patients exhibited significantly higher TNF-α levels and significantly reduced HLA-DR expression on intermediate monocytes compared with CM− patients.

**Conclusions:**

This exploratory pilot study suggests that childhood maltreatment may be associated with an altered inflammatory profile in breast cancer patients. Larger prospective studies are warranted to confirm these findings and determine their clinical significance.

## Highlights

• Childhood maltreatment is linked to elevated TNF-α in breast cancer patients.
• Reduced monocyte HLA-DR in breast cancer patients with maltreatment history.
• Psychosocial history shapes inflammatory profiles in women with breast cancer.

## 1 Introduction

In Colombia, child maltreatment remains a major public health concern. According to the Instituto Colombiano de Bienestar Familiar (ICBF), by February 2025, 71,148 children and adolescents were undergoing the administrative process of rights restoration, with sexual violence (n = 19,942) and neglect or omission (n = 19,716) being the primary reasons for their placement, followed by physical violence (n = 2085) and psychological violence (n = 1163) ((ICBF) IC and dBF, 2025). The Sivigila surveillance system reported 158,313 suspected cases of gender-based and domestic violence in 2023, of which 14.9% involved neglect and abandonment ((INS) IN and dS, 2024). In this context, a substantial proportion of Colombian children and adolescents are exposed to maltreatment, and childhood maltreatment has been associated with persistent depressive symptoms throughout the lifespan, as well as anxiety disorders and self-harm, conditions that globally account for the largest share of disease burden among survivors of childhood sexual violence (Collaborators GIPVaSVaC, 2026). The Colombian National Mental Health Survey 2015 (ENSM-2015) classifies childhood maltreatment and sexual abuse as critical life events that exceed individual adaptive capacity and generate persistent mental health suffering (Gómez-Restrepo CRMNea, 2015). Accordingly, the ENSM-2015 reports a 12-month prevalence of major depressive disorder of 1.6% in Colombia (2.3% in women; 0.9% in men), with a lifetime prevalence of 4.3% (Gómez-Restrepo CRMNea, 2015). Physiological and biomolecular studies in recent years have established a relationship between early-life exposure to chronic stress and changes in the development of the nervous, endocrine, and immune systems, which results in impaired cognitive, social, and emotional functioning and increased chronic physiological damage (Hughes et al., 2017).

A history of child abuse has been associated with immune system dysregulation, mainly characterized by elevated levels of pro-inflammatory cytokines and accelerated immune aging (immunosenescence) (Trintinaglia et al., 2018). This immune dysregulation is thought to arise, at least in part, from the long-term effects of early adversity on the hypothalamic-pituitary-adrenal (HPA) axis. Adults with a history of childhood stress exhibit heightened HPA reactivity, including altered cortisol responses to psychosocial stressors (Hughes et al., 2017), which may accelerate immune aging through chronic glucocorticoid exposure and sustained inflammatory signaling. This process is reflected in telomere shortening and shifts in specific T cells subpopulations, including the expansion of terminally differentiated T cells. Furthermore, there is increased expression of immune exhaustion markers such as PD-1 (programmed cell death protein 1), TIM-3 (T cell immunoglobulin and mucin domain 3), CTLA-4 (cytotoxic T-lymphocyte-associated protein 4), and LAG-3 (lymphocyte activation gene 3) on effector T cells. These cells, although retaining CD28 expression, lose the ability to secrete cytokines in response to antigens (Crespo et al., 2013).

Immunosenescence is further accompanied by a chronic low-grade inflammatory state termed inflammaging, characterized by persistently elevated circulating levels of pro-inflammatory cytokines, particularly IL-6 and TNF-α (Franceschi et al., 2007). This inflammatory milieu has been associated with tumor-promoting effects across several malignancies (Franceschi et al., 2017) and may specifically contribute to breast cancer development and progression by favoring a microenvironment permissive to malignant transformation (Cruz-Reyes et al., 2023-03). The relationship between immunosenescence and cancer susceptibility, however, is complex. While impaired immunosurveillance may allow tumor escape, evidence also suggests that immune-mediated inflammatory signals can actively drive tumor growth (de Magalhaes, 2013).

Additionally, senescence is characterized by cell cycle arrest and the progressive loss of surface costimulatory molecules such as CD28 and CD27 (Z et al., 2023). This process is accompanied by the upregulation of markers like KLRG-1 and CD57, as well as cell cycle regulatory proteins such as p16 and p21 (Rodríguez et al., 2020). In this context, breast cancer patients with a history of childhood maltreatment may exhibit features of accelerated immune aging, including increased expression of senescence markers (CD57 and KLRG1), immune exhaustion markers (PD-1, TIM-3, LAG-3, and CTLA-4), altered monocyte activation (HLA-DR), and dysregulated NK cell receptor expression (NKp30 and NKG2D). These alterations could compromise antitumor immune surveillance and potentially contribute to tumor progression (Rodríguez et al., 2023).

Today, it is widely accepted that immunosenescence, beyond increasing susceptibility to infections, contributes to greater cancer- and autoimmunity-related morbidity and mortality with advancing age (Lian et al., 2020). Consistent with a link between cancer, aging, and cellular senescence, the incidence of breast cancer rises steadily from around 45 years of age (Rodríguez et al., 2023). Worldwide, breast cancer is the most commonly diagnosed cancer and the leading cause of cancer death in women (Bray et al., 2024). In Colombia, it is likewise the most frequent cancer among women, with an incidence in 2018 of 13,380 new cases per year and persistently high mortality (Wiesner-Ceb et al., 2020). Within this context, this study aimed to evaluate the expression of exhaustion and senescence markers in immune cells from breast cancer patients, as well as to explore their possible association with a history of child maltreatment.

## 2 Materials and methods

### 2.1 Patients and blood samples

The study was conducted in accordance with the Declaration of Helsinki on Ethical Principles for Medical Research Involving Human Subjects (Association, 2013). It was approved by the Ethics Committee of the Instituto Nacional de Cancerología, Bogotá, Colombia (reference number ACT-043 No, 2018). Eligible participants were adult women with histologically confirmed primary invasive ductal or lobular breast carcinoma, who were scheduled to receive neoadjuvant chemotherapy with doxorubicin and cyclophosphamide as first-line treatment, had a Karnofsky Performance Status >70% or an ECOG performance status of 0–1, and provided written informed consent. Exclusion criteria included previous oncological treatment (radiotherapy, chemotherapy, immunotherapy, or gene therapy); mammary sarcoma, lymphoma, or metaplastic carcinoma; active autoimmune disease requiring treatment; evidence of acute or chronic infection (including HIV, viral hepatitis, or tuberculosis); treatment with systemic immunosuppressive agents, including corticosteroids or drugs such as azathioprine, prednisone, or cyclosporine A, within 4 weeks before enrollment (topical, intranasal, or inhaled corticosteroids were permitted); clinically significant cardiomyopathy; and previous splenectomy. These criteria were established to minimize potential confounding effects on immune and inflammatory profiles.

A total of 31 women were invited to participate. Of these, 20 agreed to donate peripheral blood samples and complete the Childhood Trauma Questionnaire–Short Form (CTQ-SF) (Bernstein et al., 2003; Hernandez et al., 2013). All participants were newly diagnosed and had not initiated systemic therapy at the time of blood collection. Peripheral blood mononuclear cells (PBMCs) were isolated from whole blood using a Histopaque density gradient (Sigma Aldrich) and cryopreserved in a solution of AIM-V 50% (Gibco, ThermoFisher Scientific), fetal bovine serum (FBS) 40% (Gibco, ThermoFisher Scientific), and dimethyl sulfoxide (DMSO) 10% (MP Biomedicals, LLC) in liquid nitrogen until use.

### 2.2 Assessment of childhood maltreatment

All 20 participants completed the Childhood Trauma Questionnaire – Short Form (CTQ-SF) (Bernstein et al., 2003), in its validated Spanish version (Hernandez et al., 2013), a retrospective self-report instrument widely validated for the detection of adverse childhood experiences. The CTQ-SF includes 28 items that evaluate five domains of childhood maltreatment: emotional abuse, physical abuse, sexual abuse, emotional neglect, and physical neglect. Subscale scores were classified into four severity levels according to the cutoff scores established in the CTQ manual (Bernstein, 1998). The questionnaire was administered through structured interviews conducted by a final-year psychiatry resident trained in clinical interviewing and trauma-informed approaches.

### 2.3 Flow cytometry

Peripheral blood mononuclear cells (PBMCs) from each study participant were thawed and washed in AIM-V medium (Gibco, ThermoFisher) prior to use in all experiments. For all assays, PBMCs were handled under sterile conditions and stained on ice or at 4°C in the dark to preserve marker integrity. For direct *ex-vivo* analysis, 1x10^6^ PBMCs were stained in 50 μL of PBS with monoclonal antibodies targeting monocyte and NK cell markers. The staining panels included: Monocytes (BioLegend): FITC anti-CD14 (clone M5E2), PE/Cy5 anti-CD16 (clone 3G8), PE/Dazzle594 anti-HLA-DR (clone L243), and APC anti-PD-L1 (clone 29E.2A3). NK Cells (BioLegend): Pacific Blue™ anti-CD3, FITC anti-CD56 (clone 5.1H11), APC anti-CD57 (clone HNK-1), APC/Fire750 anti-KLRG1 (clone SA231A2), PE/Cy7 anti-NKp30 (clone P30-15), and PE anti-NKG2D (clone 1D11).

CD14 and CD16 were used to identify the three peripheral blood monocyte subpopulations: classical (CD14^++^CD16^−^), intermediate (CD14^++^CD16^+^), and non-classical (CD14low CD16^+^). HLA-DR was included to assess antigen-presenting capacity, as decreased MHC class II expression is a recognized feature of monocyte immune aging (Nyugen et al., 2010). PD-L1 was assessed as an immune checkpoint marker whose expression on monocytes has been associated with immunosuppression through inhibition of T cell activation via PD-1 binding (Nagano et al., 2018/11). NK cells were identified by CD56 and CD16 expression and absence of TCR. Based on CD56/CD16 co-expression, three subpopulations were distinguished: CD56^bright^ CD16^−^, CD56^dim^ CD16^+^, and CD56^neg^ CD16^+^ (Tarazona et al., 2015). CD57 and KLRG-1 were included as markers of NK cell maturation and senescence (Campos et al., 2014). NKp30 and NKG2D were assessed as activating receptors critical for tumor cell recognition and initiation of cytotoxic activity (Campos et al., 2014).

To assess exhaustion and senescence in T cells, 1x10^6^ PBMCs per well were seeded into 96-well flat-bottom plates and stimulated for 72 h with anti-CD3/CD28/CD2 antibody-coated beads (Miltenyi Biotec) at a 2:1 PBMC-to-bead ratio, using AIM-V medium (Thermo Fisher Scientific). After incubation, cells were washed with PBS and stained with the following monoclonal antibodies: BioLegend: Pacific Blue™ anti-CD3, Brilliant Violet 510™ anti-CD4 (clone SK3), PE/Dazzle594 anti-CD8 (clone SK1), FITC anti-CD45RO (clone UCHL1), PerCP/Cy5.5 anti-PD-1 (clone EH12.2H7), PE/Cy7 anti-CTLA-4 (clone L3D10), PE anti-TIM-3 (clone F38-2E2), and APC/Fire™750 anti-KLRG1 (clone SA231A2). BD Biosciences: BV711 anti-CD62L (clone DREG-56), APC anti-CD57 (clone NK-1), and BV786 anti-LAG-3 (clone T47-530). Cells were incubated for 20 min in the dark at room temperature, washed with PBS, and resuspended in FACS Flow buffer. At least 100,000 events were acquired per sample using a FACSAria III flow cytometer (BD Biosciences). Data were analyzed with FlowJo™ software v10 (BD Biosciences).

The T cell panel was designed to simultaneously evaluate immunosenescence and immune exhaustion, two related but mechanistically distinct processes that compromise T cell function (Akbar and Henson, 2011). Marker selection was based on a prior systematic review by our group (Rodríguez et al., 2020), which identified increased expression of CD57 and KLRG-1 in effector T cell subsets as the primary flow cytometry markers of senescent T cells. Immune exhaustion was evaluated through the expression of PD-1, CTLA-4, TIM-3, and LAG-3 (Crespo et al., 2013).

**Supplementary Fig. 1:**
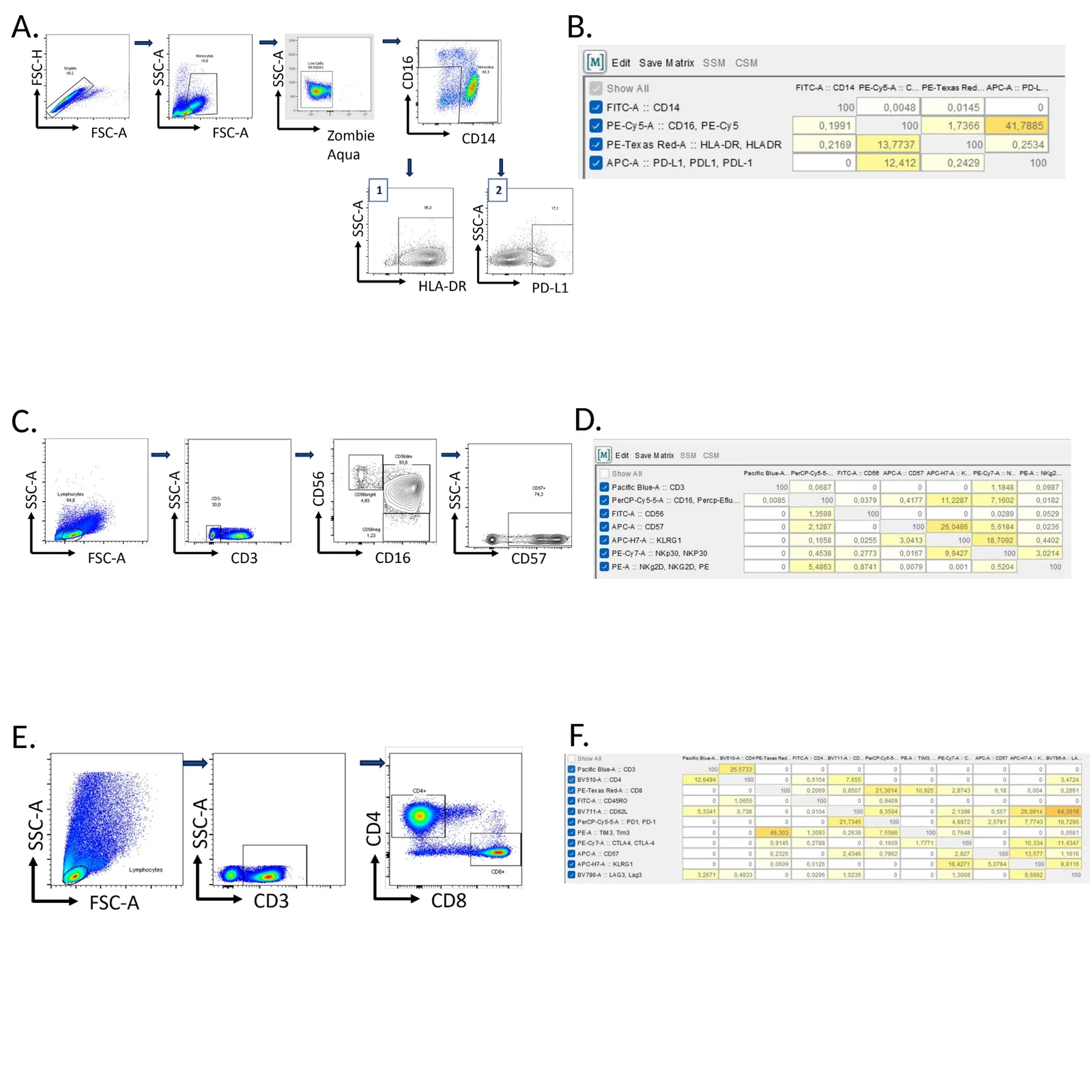
Representative flow cytometry gating strategies and compensation matrices for the monocyte, NK-cell, and T-cell panels. Sequential gating strategies are shown for (A) monocyte subsets, identified by CD14 and CD16 expression, with subsequent assessment of HLA-DR and PD-L1; (C) NK-cell subsets, defined by CD16 and CD56 expression, with assessment of the senescence marker CD57; and (E) CD4^+^ and CD8^+^ T-cell subsets. Automatically generated fluorescence compensation matrices are shown for the (B) monocyte, (D) NK-cell, and (F) T-lymphocyte panels; these matrices were produced by the BD FACSAria III flow cytometer following the instrument's standard compensation procedure using the appropriate single-stained controls. Cell viability was assessed before antibody staining by trypan blue exclusion and cell counting, and exceeded 90% in all experiments.

Cell populations were identified using sequential gating strategies based on forward- and side-scatter (FSC/SSC) characteristics, followed by singlet discrimination and viability gating. Fluorescence compensation was performed before sample acquisition using single-stained BD™ CompBeads (BD Biosciences) for each fluorochrome included in the corresponding antibody panel. Data were acquired across multiple experimental runs, and a reference control sample was included in each run to monitor inter-run variability. All data were analyzed using FlowJo software (version 10.1; BD Biosciences). Representative gating strategies for each panel are shown in Supplementary Fig. S1.

### 2.4 Cytokine quantification by CBA assay

Plasma samples from patients were used to quantify inflammatory cytokines using the Human Th1/Th2 Cytokine Kit II (BD Biosciences), which allows the simultaneous measurement of IL-2, IL-4, IL-6, IL-10, TNF-α, and IFN-γ. The assay was performed according to the manufacturer's instructions. Briefly, samples were incubated with beads coated with specific capture antibodies for each cytokine. After washing, the beads were stained with a PE-conjugated detection antibody. Samples were acquired on a FACSAria III flow cytometer (BD Biosciences), and cytokine concentrations were calculated using the BD CBA analysis software, based on standard curves generated for each analyte. Samples were analyzed as single measurements (singlets). No sample dilution was required. Assay performance was verified by inclusion of an 8-point standard curve (20–5000 pg/ml) in each run per manufacturer's protocol; the kit reports intra-assay CVs ≤5% and inter-assay CVs ≤11% for all measured cytokines (Biosciences et al., 2026).

### 2.5 Statistical analysis

Statistical analyses and graphs were performed using Prism version 9 (GraphPad Software). Comparisons between groups (positive vs. negative history of childhood maltreatment) were conducted using non-parametric tests: the Mann–Whitney *U* test for two-group comparisons and the Kruskal–Wallis test for multiple-group comparisons when applicable. A p-value < 0.05 was considered statistically significant. Given the exploratory nature of this study and the limited sample size, no adjustment for multiple comparisons was performed. Therefore, the findings should be considered hypothesis-generating and require confirmation in larger, adequately powered cohorts.

## 3 Results

### 3.1 Sociodemographic and clinical characteristics

The sociodemographic and clinical characteristics of the sample are summarized in Table 1. All participants were women diagnosed with breast cancer, with a mean age of 52.95 years and an average weight of 66.9 kg. 70% of the participants were from the Andean region, and 50% reported being single. Additionally, 15% (n = 3) had a history of prior psychiatric evaluation, defined as having previously required psychiatric care at any point in their life; a borderline association was observed between this variable and a history of sexual abuse (p = 0.07), which should be interpreted cautiously given the limited sample size. Regarding clinical status, most patients presented with invasive ductal carcinoma, with histological grades G2 or G3. The Karnofsky Performance Status Index was ≥ 90 in 85% of cases, indicating preserved functional status at the time of evaluation. As for histological biomarkers, 35% of the patients were HER2-positive, while the expression of hormone receptors was heterogeneous, with a higher frequency of estrogen and progesterone receptor positivity. Tumor proliferative activity, assessed by the Ki67 expression index, showed a median of 50% (range 8–100%), with 70% of patients (n = 14) exhibiting values above 20%, indicative of high tumor proliferative activity. Ki67 levels were comparable between the groups.

**Table 1:** Demographic and clinical data of participants.

	CM-	CM+	Total	p -value
**Age**	53.8 ± 7.8	52.1 ± 11	52.95 ± 9.3	0.940
**Weight**	68.9 ± 12.4	64.9 ± 13.2	66.9 ± 12.7	0.597
**Karnofsky**	>94%	>95%	94,5	N/A
**Status**				
**IIA**	4	2	6	N/A
**IIIA**	0	2	2	N/A
**IB**	0	1	1	N/A
**IIB**	2	0	2	N/A
**IIIB**	4	5	9	N/A
**HER2**				
**Positive**	1	3	4	0.582^a^
**PR (%)**				
**Positive**	7 (1-100%)	8 (10-100%)	15	1
**ER (%)**				
**Positive**	7 (60-100%)	7 (5-100%)	14	1
**Ki67 (%)**	50 ± 31 (%)	55 ± 34 (%)	52 ± 32	0.650
**CTQ**	36.5 ± 8.7	68 ± 13.7	52.25 ± 19.7	N/A

a CM: Childhood Maltreatment; HER2: Human Epidermal Growth Factor Receptor 2, PR: Progesterone Receptor; ER: Estrogen Receptor, CTQ: Childhood Trauma Questionnaire–Short Form. Data are expressed as mean ± SD or frequency (n). CM−: patients without history of childhood maltreatment (CTQ total score ≤50); CM+: patients with history of childhood maltreatment (CTQ total score >50).

### 3.2 Severity of childhood maltreatment across CTQ-SF

Subscale severity was classified according to established cutoffs (Bernstein, 1998) as None or Minimal, Low to Moderate, Moderate to Severe, or Severe to Extreme. Table 2 shows the severity shows the severity distribution across the five CTQ-SF subscales for the full sample (n = 20) (Hernandez et al., 2013). Within this sample, 40% (n = 8) reported severe-to-extreme physical abuse, 35% (n = 7) severe-to-extreme emotional abuse, and 40% (n = 8) severe-to-extreme physical neglect. Regarding sexual abuse, 60% (n = 12) reported no history, while the remaining 40% were distributed across low-to-extreme severity levels. These findings reflect a high prevalence of adverse childhood experiences in this population of women with breast cancer.

**Table 2:** Frequency of childhood abuse by category in patients assessed with the CTQ-SF.

Category	None (or Minimal)	Low (to Moderate)	Moderate (to Severe)	Severe (to Extreme)
**Emotional Abuse**	10	2	1	7
**Physical Abuse**	8	1	3	8
**Sexual Abuse**	12	3	2	3
**Emotional Neglect**	8	5	4	3
**Physical Neglect**	7	3	2	8

CTQ-SF: Childhood Trauma Questionnaire–Short Form. Severity categories: None or minimal, Low to moderate, Moderate to severe, and Severe to extreme. Values represent number of patients (n = 20).

### 3.3 Immune phenotypes are largely preserved in breast cancer patients with a history of childhood maltreatment

Immunological markers and cellular populations were compared between breast cancer patients who reported a history of childhood maltreatment (CM+, n = 10; CTQ total score >50) and those who did not (CM−, n = 10; CTQ total score ≤50). In terms of T cells immunophenotype, no statistically significant differences were observed in the frequency of CD4^+^ or CD8^+^ cells, nor in the expression of exhaustion or senescence markers (PD-1, CTLA-4, TIM-3, LAG-3, KLRG1, CD57). However, non-significant tendencies were identified, including higher KLRG1 expression in CD8^+^ cells from the abused group (22.51 ± 19.16 vs. 13.83 ± 11.74; p = 0.24), and lower CTLA-4 expression in CD8^+^ cells in the same group (3.16 vs. 10.59; p = 0.089) (Table 3).

**Table 3:** Immunophenotyping of major lymphocyte subsets.

Variable	CM- (n = 10)	CM+ (n = 10)	p-value	Statistical Test
**CD3+**	71.82 ± 9.44	67.18 ± 11.11	0.328	*t*-test
***CD4 T cells***				
**CD4+**	62.20 ± 14.76	66.18 ± 6.90	0.4538	*t*-test
**CD4+/CD57+**	2.33 (0.81–7.99)	2.98 (0.43–5.75)	0.7913	Mann–Whitney
**CD4+/CTLA-4+**	5.29 (0.98–9.02)	1.75 (0.97–3.52)	0.3447	Mann–Whitney
**siCD4+/KLRG1+**	3.65 (1.81–21.68)	4.17 (0.94–50.85)	0.8501	Mann–Whitney
**CD4+/LAG3+**	6.91 (4.81–60.90)	6.45 (3.79–79.03)	0.9097	Mann–Whitney
**CD4+/PD-1+**	2.37 (1.07–7.12)	2.87 (1.00–7.12)	0.9097	Mann–Whitney
**CD4+/TIM3**	18.16 ± 14.12	20.24 ± 9.56	0.7043	*t*-test
***CD8 T cells***				
**CD8^+^ T cells**	27.41 ± 12.01	24.34 ± 7.91	0.51	*t*-test
**CD8+/CD57+**	21.00 ± 20.93	17.32 ± 13.18	0.6448	*t*-test
**CD8+/CTLA-4+**	10.59 (3.59–20.57)	3.16 (1.84–9.24)	0.089	Mann–Whitney
**CD8+/KLRG1+**	13.83 ± 11.74	22.51 ± 19.16	0.2412	*t*-test
**CD8+/LAG3+**	48.15 (12.93–52.98)	47.85 (16.40–61.98)	0.5205	Mann–Whitney
**CD8+/PD-1+**	3.87 (1.48–6.42)	4.75 (1.64–14.61)	0.5708	Mann–Whitney
**CD8+/TIM3**	28.70 (7.77–34.62)	28.05 (12.55–33.55)	1.0	Mann–Whitney
***NK cells***				
**NK cells**	12.07 ± 7.93	11.90 ± 6.92	0.9613	*t*-test
**NK cells/CD57+**	31.44 ± 19.63	38.61 ± 16.06	0.3835	*t*-test
**NK cells/KLRG1+**	30.09 ± 26.29	37.32 ± 22.49	0.5176	*t*-test
**NK cells/NKG2D+**	73.95 (68.55–74.70)	77.25 (69.40–80.67)	0.1617	Mann–Whitney
**NK cells/NKp30+**	35.35 ± 24.01	34.68 ± 19.49	0.9465	*t*-test
**CD56dim**	80.78 ± 8.50	81.61 ± 6.38	0.8079	*t*-test
**CD56bright**	2.83 (2.48–5.30)	6.01 (4.88–8.16)	0.1508	Mann–Whitney
**CD56neg**	14.02 ± 8.96	11.83 ± 6.33	0.5368	*t*-test
***Monocytes***				
**Monocytes**	11.33 ± 3.43	13.65 ± 5.71	0.2891	*t*-test
**Monocytes HLADR+**	92.80 (86.47–97.53)	91.20 (88.78–94.65)	0.5708	Mann–Whitney
**Monocytes PDL1+**	18.90 (12.22–23.97)	12.95 (9.19–21.35)	0.3845	Mann–Whitney
**Classical**	73.47 ± 9.90	77.79 ± 9.81	0.34	*t*-test
**Classical HLADR+**	95.30 (86.00–99.12)	92.20 (86.45–97.92)	0.623	Mann–Whitney
**Classical PDL1+**	2.63 (1.27–5.28)	1.38 (0.95–3.45)	0.4274	Mann–Whitney
**Intermediate**	15.40 (12.20–18.68)	13.20 (10.64–21.07)	0.5452	Mann–Whitney
**Intermediate HLADR+**	94.95 (91.75–97.58)	86.25 (82.90–89.42)	**0.0452**	Mann–Whitney
**Intermediate PDL1+**	76.00 (27.32–90.97)	65.25 (15.80–95.28)	0.9697	Mann–Whitney
**Non-classical**	7.03 (3.77–7.81)	5.18 (4.75–5.77)	0.3845	Mann–Whitney
**Non-classical HLADR+**	49.89 ± 24.93	39.00 ± 22.94	0.3229	*t*-test
**Non-classical PDL1+**	95.15 (40.90–99.65)	50.45 (10.62–91.00)	0.2247	Mann–Whitney

CM−: patients without a history of childhood maltreatment (n = 10); CM+: patients with a history of childhood maltreatment (n = 10). Data are expressed as mean ± SD for normally distributed variables or median (interquartile range) for non-normally distributed variables. Statistical comparisons were performed using Student's t-test or Mann–Whitney *U* test, as appropriate. Bold p-value indicates statistical significance (p < 0.05). NK: natural killer; HLA-DR: human leukocyte antigen DR; PD-L1: programmed death-ligand 1; KLRG1: killer cell lectin-like receptor G1; LAG-3: lymphocyte activation gene 3; TIM-3: T cell immunoglobulin and mucin domain 3; PD-1: programmed cell death protein 1; CTLA-4: cytotoxic T-lymphocyte-associated protein 4.

Regarding NK cells, no significant differences were found in the overall frequency of NK cells, their subpopulations (CD56^bright^, CD56^dim^, CD56^neg^), or in the expression of markers such as CD57, KLRG1, NKp30, and NKG2D. Similarly, PD-L1 levels in monocytes did not show significant differences between groups. However, a significant decrease in HLA-DR expression on intermediate monocytes was observed in patients with a history of abuse (86.25% vs. 94.95%; p = 0.0452), suggesting reduced surface expression of HLA-DR, a marker associated with antigen-presenting capacity, in this monocyte subpopulation (Table 3).

### 3.4 Elevated TNF-α levels in patients with childhood maltreatment history

**Fig. 1:**
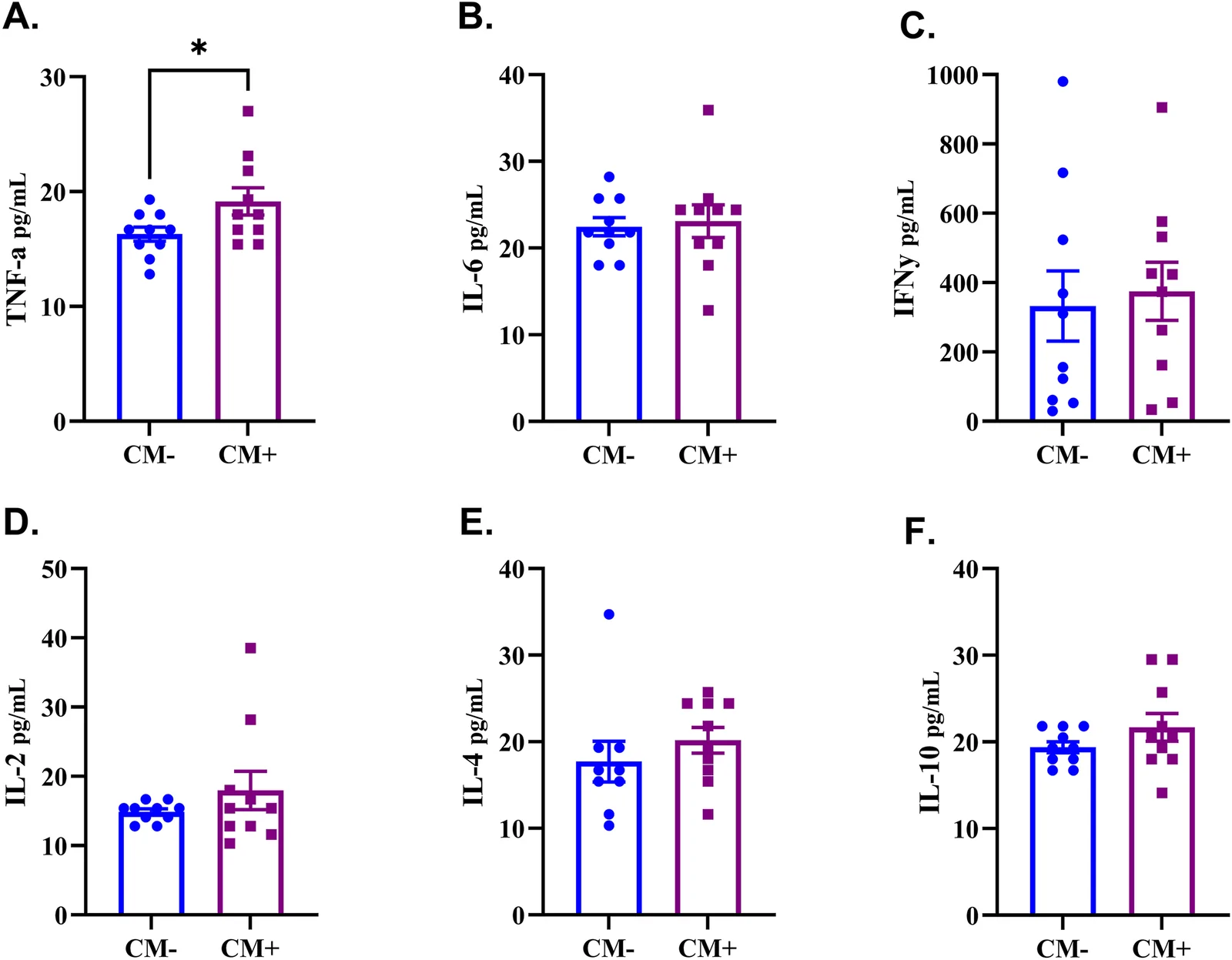
**Plasma cytokine concentrations by group.** Bar graphs show mean ± SEM for (A) TNF-α, (B) IL-6, (C) IFN-γ, (D) IL-2, (E) IL-4, and (F) IL-10 (n = 10 per group). CM−: patients without history of childhood maltreatment; CM+: patients with history of childhood maltreatment. *p < 0.05 (Mann–Whitney *U* test, one-tailed)**.**

A statistically significant difference was observed in TNF-α levels, which were higher in the CM + group (19.14 ± 3.76 pg/mL) compared to CM− (16.31 ± 1.94 pg/mL; U = 27.5, p = 0.046) (Fig. 1). No significant differences were found between groups for the other evaluated cytokines (IFN-γ, IL-10, IL-6, IL-2, and IL-4). However, wide interindividual variability was observed, particularly in IFN-γ (CM+: CV = 70%; CM−: CV = 97%) and IL-2 (CM+: CV = 49%) concentrations, which may have contributed to reduced statistical power for detecting between-group differences in these cytokines. These findings support the hypothesis of low-grade immunological dysregulation in patients exposed to childhood abuse, with TNF-α emerging as a potential biomarker to be explored in future studies.

## 4 Discussion

Child maltreatment refers to abuse and neglect affecting children under the age of 18; it includes all forms of physical and/or emotional mistreatment, sexual abuse, neglect, and commercial or other types of exploitation that result in actual or potential harm to health (Cruz-Reyes et al., 2023-03). In Colombia, the high prevalence of childhood maltreatment highlights the importance of investigating its potential impact on immune function. In the present study, we explored whether a history of childhood maltreatment was associated with inflammatory and immunological alterations in women with breast cancer.

In our sample (n = 20), analysis of the CTQ-SF questionnaire revealed a high burden of adverse childhood experiences: 40% of patients reported severe physical abuse, 35% reported severe emotional abuse, and 40% reported severe physical neglect. Although 60% denied a history of sexual abuse, it is important to consider possible false negatives related to the minimization/denial items included in the scale. Regarding the relationship between sociodemographic variables and maltreatment domains, those with a history of sexual abuse tended to have previously received psychiatric care, which is consistent with the literature linking this type of trauma to higher rates of mental disorders in adulthood and a greater risk of developing cancer (Brown et al., 2013; Gerhardt et al., 2022; Herrenkohl et al., 2021; Hu et al., 2021). These findings are concerning, especially considering the small sample size, and are consistent with national reports indicating a high rate of child maltreatment in Colombia. These data highlight the importance of exploring the impact of early adverse experiences on the physical, mental, and immunological health of adult women, particularly in clinical contexts such as cancer.

Several studies have shown that childhood exposure to chronic stress leads to alterations in the development of the nervous, endocrine, and immune systems, resulting in impaired cognitive, social, and emotional functioning, as well as increased chronic physiological damage (Hughes et al., 2017; Wieck et al., 2014; Smith, 2020). These changes are evident in adults with a history of maltreatment as heightened reactivity to stressors, abnormal hypothalamic-pituitary-adrenal (HPA) axis responses, and sustained increases in the production of proinflammatory cytokines such as TNF-α, IL-6, IL-1β, and IFN-γ (Wieck et al., 2014; Smith, 2020; Passos et al., 2015; Murphy et al., 2025). These conditions promote a state of low-grade chronic inflammation, partly mediated by activation of the transcription factor NF-κB (Pace et al., 2012), which has been linked to immune dysfunction, glucocorticoid resistance in monocytes, and accelerated immunological aging.

In our study, we observed that breast cancer patients with a history of childhood maltreatment had significantly higher plasma levels of TNF-α compared to those without such history. This finding is consistent with the literature describing an exacerbated inflammatory axis in individuals exposed to early-life trauma (Passos et al., 2015; Brown et al., 2021). For example, elevated TNF-α has been reported in older adults with a history of childhood maltreatment (Hu et al., 2021; Carpenter et al., 2010), as well as in caregivers (Kiecolt-Glaser et al., 2011). A meta-analysis also demonstrated increased levels of molecules such as TNF-α, IL-6, and CRP, supporting the findings of this study (Baumeister et al., 2016).

Elevated TNF-α levels have been associated with immune exhaustion in both innate and adaptive immune cells, as it induces epigenetic and transcriptional changes that lead to suboptimal activation of these cells (Wang et al., 2022). Furthermore, increased serum TNF-α has also been linked to insulin resistance, which contributes to the development of metabolic and cardiovascular diseases (Tylutka et al., 2024). Altogether, these findings support the notion of low-grade chronic inflammation, a condition that promotes tumor development, is present in individuals that experience childhood maltreatment.

Although no statistically significant differences were observed in the expression of senescence or exhaustion markers on T cells or NK cells, numerical trends were noted, including a higher proportion of KLRG1^+^ CD8^+^ T cells in the CM+ group. Given the exploratory design and limited sample size, these findings should be interpreted with caution and do not provide evidence of altered immune function without complementary functional studies. Whether these phenotypic trends have biological or clinical relevance remains to be determined in larger, adequately powered cohorts.

A previous study by Trintinaglia et al. in breast cancer patients reported a reduction in naïve T cells and an increase in terminally differentiated T cells in patients with a history of maltreatment, as well as higher expression of senescence markers (KLRG1, CD57) compared to those who did not report such experiences (Trintinaglia et al., 2018). Although we observed similar numerical trends, these differences did not reach statistical significance and should be interpreted cautiously given the exploratory nature of the study. Larger studies are needed to determine whether these patterns are reproducible.

Additionally, a significant reduction in HLA-DR expression on intermediate monocytes was observed in patients with a history of childhood maltreatment. While reduced HLA-DR expression has been reported in association with altered monocyte function in other clinical settings (Mengos et al., 2019), no functional assays were performed in the present study. Therefore, the biological significance of this finding in our cohort remains uncertain and warrants further investigation. It has also been noted that in early-stage breast cancer patients, childhood maltreatment was associated with chronic immune activation, especially among those with depressive symptoms, which could impact long-term health and recovery (Bower et al., 2020).

Taken together, the findings of this exploratory pilot study suggest that childhood maltreatment may be associated with subtle immunological differences in patients with breast cancer, particularly with respect to TNF-α levels and HLA-DR expression on intermediate monocytes. Although no significant associations were observed between the severity of childhood maltreatment and the immune markers evaluated, the high prevalence of reported maltreatment in this cohort, consistent with childhood maltreatment being recognized at the national level as a prevalent adverse childhood experience in Colombia (ENSM-2015) (Gómez-Restrepo CRMNea, 2015), reinforces the relevance of considering early-life psychosocial adversity in breast cancer research and of incorporating psychosocial screening into comprehensive oncological care. Larger, adequately powered cohorts are needed to determine the reproducibility and clinical relevance of these preliminary findings and to further explore the relationship between childhood maltreatment and cancer-related immune responses.

## 5 Limitations

First, the relatively small sample size reduced the statistical power to detect significant associations between childhood maltreatment and immune variables. In addition, the high dimensionality of the immunological analyses relative to the sample size increased the risk of false-positive findings. Because of the exploratory nature of this study, no adjustment for multiple comparisons was performed. Therefore, the findings should be considered hypothesis-generating and require confirmation in larger, adequately powered cohorts.

A second limitation is the lack of a psychometrically validated Colombian version of the Childhood Trauma Questionnaire–Short Form (CTQ-SF). Therefore, we used the Spanish version validated by Hernández et al. (Hernandez et al., 2013). This study did not control for potential confounders such as current stress, depressive symptom severity, BMI, menopausal status, or lifestyle factors, which may independently influence immune senescence and exhaustion markers. Future studies should incorporate these variables to better isolate the contribution of childhood maltreatment history.

## CRediT authorship contribution statement

**Ivon Johanna Rodríguez-Rodríguez:** Writing – review & editing, Writing – original draft, Validation, Supervision, Methodology, Investigation, Formal analysis, Conceptualization. **Sara Lucía Neira-Ortíz:** Writing – original draft, Investigation. **Manuela Llano-León:** Writing – original draft, Methodology, Investigation. **Nicolas Lalinde-Ruiz:** Writing – review & editing, Methodology, Investigation. **Henry Mauricio Parada-Gereda:** Writing – review & editing, Methodology, Formal analysis. **Ricardo Sanchez-Pedraza:** Writing – original draft, Formal analysis, Conceptualization. **Carlos Alberto Parra-López:** Writing – review & editing, Writing – original draft, Validation, Supervision, Project administration, Funding acquisition, Conceptualization.

## Ethics declaration

Written informed consent to take part in the study and to publish the article has been obtained from all participants or their legal representatives. The privacy rights of participants have been observed.

This study included organ or tissue donors. This study includes human biological material and consent was obtained by donors, or their next of kin or legal representatives, for use in this study and for publication of the article. The samples used in this research were not sourced from executed prisoners or prisoners of conscience.

This study was performed in compliance with relevant laws, regulatory frameworks and guidelines where the research took place. This study was conducted in accordance with Resolución No. 008430 de 1993 por la cual se establecen las “Normas Científicas, Técnicas y Administrativas para la Investigación en Salud. This study was approved by the Instituto Nacional de Cancerología (Colombia). (Approval No. ACT-043 No, 2018)

## Declaration of generative AI and AI-assisted technologies in the manuscript preparation process

During the preparation of this work the authors used Claude and Grammarly in order to improve English language clarity and grammar editing. After using this tool/service, the authors reviewed and edited the content as needed and takes full responsibility for the content of the published article.

## Funding

This work was supported by the División de Investigación Sede Bogotá, 10.13039/501100002753Universidad Nacional de Colombia (Código Hermes: 47334, 48357). MinCiencias (Contrato No. 920 de 2023, Contrato No. 847 de 2020).

## Declaration of competing interest

The authors declare that they have no known competing financial interests or personal relationships that could have appeared to influence the work reported in this paper.

## Data Availability

Data will be made available on request.
